# Unerwartete Tracheale Läsion nach Intubation – Call for Discussion

**DOI:** 10.1007/s00101-025-01611-9

**Published:** 2025-11-25

**Authors:** Dovile Diktanaite, Christoph Konrad, Volker Wenzel, Roland Steinmann, Romedi Benz, Christoph M. Marggraf

**Affiliations:** 1 Department für klinische Querschnittsmedizin, Klinik für Anästhesie, Luzerner Kantonsspital, Spitalstrasse, 6006 Luzern, Schweiz; 2 Universität Luzern, Luzern, Schweiz; 3 Klinik für Anästhesie Medizin Campus Bodensee, Friedrichshafen, Deutschland; 4 Department of Anesthesiology, University of Florida, Gainesville, FL USA

## Einleitung

Trachealverletzungen können nach Thoraxtraumata oder iatrogen im Rahmen medizinischer Interventionen wie einer endotrachealen Intubation auftreten. Während Erstere häufig durch Hochgeschwindigkeitsmechanismen bedingt sind, entstehen iatrogene Läsionen meist durch mechanische oder druckbedingte Einwirkungen während der Atemwegssicherung [1].

Iatrogene Trachealverletzungen („iatrogenic tracheal injuries“, ITI) treten insbesondere im Zusammenhang mit einer Intubation, perkutaner dilatativer Tracheotomie oder starrer Bronchoskopie auf. Die Inzidenz wird auf 0,005 % aller Intubationen geschätzt. Risikofaktoren sind Notfallintubationen, Doppellumentuben, Intubationshilfen (Bougie, Führungsdraht) und Hochdruck-Cuffs. Patientenspezifische Prädispositionen umfassen anatomische Tracheaveränderungen, weibliches Geschlecht, höheres Alter, Adipositas und langfristige Steroidtherapie [2, 3]. Die klinische Präsentation reicht von asymptomatischen Verläufen über Husten, subkutanes Emphysem, Pneumomediastinum und Pneumothorax bis hin zu Mediastinitis und respiratorischer Dekompensation. Chronische Verläufe mit verzögerter Symptomatik sind ebenfalls beschrieben [2, 3].

Wir berichten über zwei Fälle von ITI nach einer störungsfreien elektiven Intubation, die von erfahrenen Anästhesisten als atraumatisch eingestuft wurden. Dennoch entwickelte eine Patientin postoperativ eine signifikante Symptomatik, die zur Diagnose einer Trachealläsion führte. Diese Fälle werfen die Frage auf, ob bislang unterschätzte Risikofaktoren, die über die bekannten Parameter hinausgehen, existieren.

## Fall 1 – schwere Tracheaverletzung

Eine 41-jährige Patientin (ASA 2, Größe 164 cm, BMI 26,8 kg/m^2^) unterzog sich ambulant einer Revisionsendodakryozystorhinostomie sowie einer Kieferhöhlenfenestrierung aufgrund einer Tränengangsstenose und rezidivierender Infekte der oberen Atemwege (chronische Sinusitis maxillaris). Die Allgemeinanästhesie mit TCI (Propofol/Remifentanil) wurde unter Aufsicht eines erfahrenen Facharztes für Anästhesie problemlos eingeleitet. Nach relaxometrisch gesteuerter Rocuroniuminjektion erfolgte die Intubation mit einem RAE-Tubus (Größe 7,5; Parker Flex-Tip® [Panyu, China]) unter direkter Laryngoskopie (Cormack-Lehane IIa) ohne Schwierigkeiten. Nach Fixierung und Cuff-Druck-Kontrolle wurde die Patientin mit rekliniertem Kopf für den endoskopischen Eingriff gelagert. Die 63-minütige Operation verlief komplikationslos. Nach gastraler und oropharyngealer Sekretabsaugung erfolgte eine problemlose Extubation. Ein initialer Hustenreiz sistierte rasch unter Laryngologikum. Die Patientin wurde 3 h 15 min später in stabilem Zustand mit leichten Halsschmerzen entlassen.

**Abb. 1:**
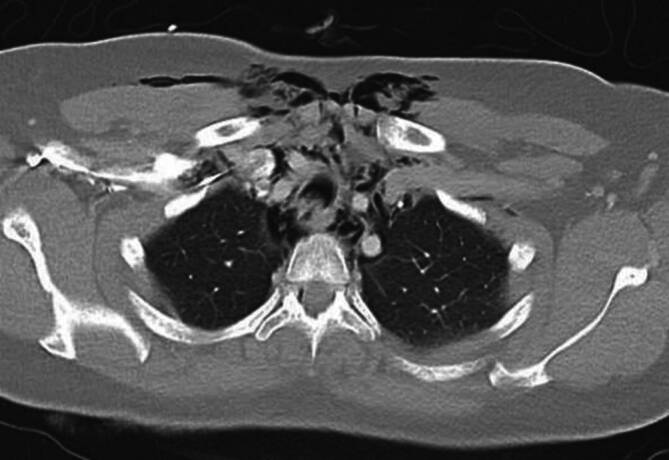
Thorax-CT bei Schockraumaufnahme: Nachweis eines ausgeprägten Weichteilemphysems mit begleitendem Pneumomediastinum

**Abb. 2:**
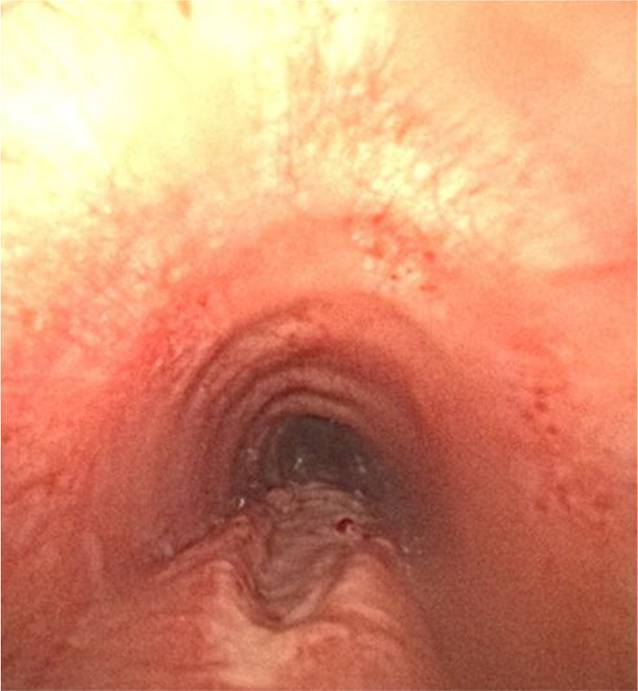
Bronchoskopie in Spontanatmung im Schockraum: langstreckige Lazeration der Trachealhinterwand

Sechs Stunden nach der Entlassung traten zunehmende Dysphagie, Luftnot und ein zervikales Weichteilemphysem auf. Die Patientin alarmierte den Rettungsdienst und wurde in den Schockraum unserer Klinik aufgenommen. Das Thorax-CT ergab den Nachweis eines ausgeprägten Weichteilemphysems mit begleitendem Pneumomediastinum sowie einem Verdacht auf eine tracheale Läsion im oberen bis mittleren Drittel (Abb. 1). Eine Bronchoskopie in Spontanatmung zeigte eine langstreckige Lazeration der Trachealhinterwand (Abb. 2); in einer Gastroskopie erfolgte der Ausschluss einer ösophagealen Perforation. Danach erfolgten die Aufnahme auf die Intensivstation, der Beginn einer empirischen antibiotischen Therapie mit Co-Amoxicillin sowie antitussiver Behandlung und die Sauerstoffgabe. Unter der Therapie bildete sich das Weichteilemphysem rasch zurück. Die Kontrollbronchoskopie zeigte eine 4 cm lange Schleimhautlazeration, die sich 3 cm median der Pars membranacea erstreckte und eine atemabhängige Trachealstenose im Sinne einer möglichen Tracheomalazie verursachte.

Nach 48 h auf der Intensivstation wurde die Patientin auf die Normalstation verlegt und am vierten Tag in gutem Zustand entlassen. Die erste bronchoskopische Nachkontrolle verlief ohne Komplikationen und ohne Anzeichen eines persistierenden Emphysems. Eine abschließende Verlaufsbronchoskopie ist nach sechs Wochen geplant.

## Fall 2 – leichtgradige Tracheaverletzung

Eine 41-jährige Patientin (Größe 165 cm, BMI 28,5 kg/m^2^) unterzog sich bei V.a. eine Sarkoidose einer elektiven Bronchoskopie. Diese war in Intubationsnarkose geplant. Die Narkoseeinleitung und -führung mittels TCI nach Klinikstandard verliefen problemlos. Der erste Intubationsversuch erfolgte ösophageal und wurde umgehend durch den Facharzt für Anästhesie ohne Einführhilfe oder spezielle Lagerung und mittels CMAC korrigiert (Standard Tracheal Tubus, Gr. 8.0, Fa. Wellead [Panyu, China]). Alle Messwerte waren im Normbereich. Der Cuff-Druck wurde standardmäßig auf 30 cm H_2_O eingestellt.

**Abb. 3:**
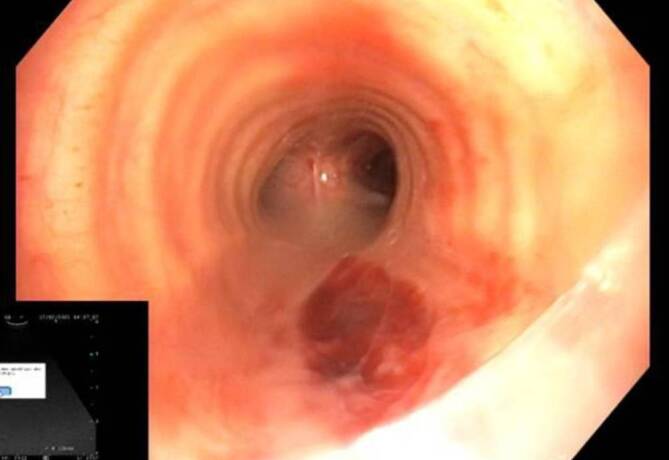
Trachealäsion in der Pars membranacea mit Schleimhautdehiszenz

**Abb. 4:**
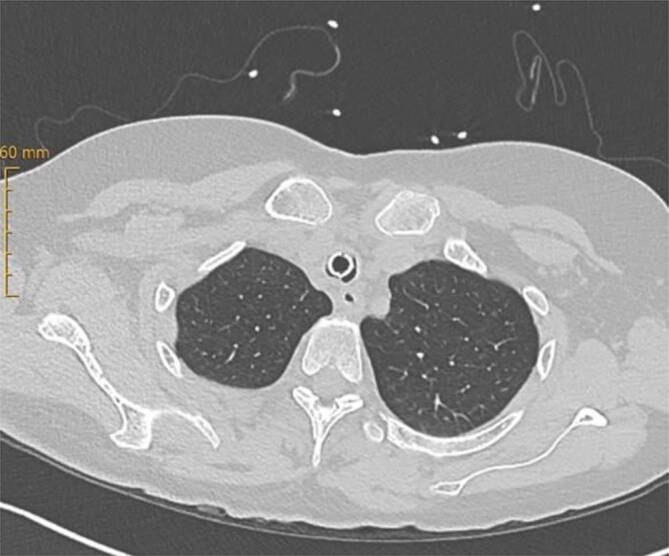
CT-Befund: Fehlen eines Luftemphysems

Im Rahmen der Bronchoskopie fiel dann zuerst eine zu tiefe Tubuslage auf und wurde korrigiert. Hierbei zeigte sich nun eine Läsion der Schleimhaut mit Dehiszenz im Bereich des mittleren Drittels der Trachea im Bereich der Pars membranacea (Abb. 3). Die genauere Inspektion, einschließlich Ultraschalldarstellung, gab keinen Hinweis für eine tiefergehende Läsion. Eine Inspektion des Ösophagus zeigte keine pathologische Veränderung. Trotzdem führten wir eine CT-Untersuchung mit der Frage nach einem Luftemphysem durch (Abb. 4); was nicht nachgewiesen werden konnte. Narkoseausleitung ohne Hustenabwehr und der weitere Verlauf waren komplikationslos. Eine endoskopische Untersuchung nach 2 Tagen zeigte eine abheilende Läsion.

## Diskussion

Tracheaverletzungen nach einer Intubation sind selten, werden jedoch aufgrund unspezifischer Symptomatik oft unterschätzt. In beiden Fällen wurde die Intubation als atraumatisch dokumentiert – im ersten Fall trat die Symptomatik nach wenigen Stunden auf, im zweiten wurde die Läsion zufällig während einer Bronchoskopie entdeckt. Die variable klinische Präsentation erschwert die Erfassung der ITI-Inzidenz. Unsere Beispiele zeigen, dass diese Komplikation auch nach elektiven, atraumatisch erscheinenden Intubationen auftreten kann.

Iatrogene Verletzungen der Trachea durch Intubation entstehen meist durch mechanische Schädigung beim Einführen des Tubus mit einem herausstehenden Führungsstab oder durch Druckschäden infolge zu hohem Cuff-Druck (< 30 cm H_2_O) [3]. Meistens wird die Pars membranacea der hinteren Trachealwand an der Mittellinie oder an der Übergangszone zwischen Knorpel und Membran auf der rechten Seite betroffen. Die Verletzungen sind typischerweise längsverlaufend und befinden sich im mittleren Drittel der Trachea [3]. Die Ausdehnung der Risse ist bis ins untere Drittel oder in die Hauptbronchien möglich [2]. Die Tiefe der Verletzung ist entscheidend für die Therapieplanung. Im Jahr 2010 entwickelten Cardillo et al. eine morphologische Klassifikation zur Risikostratifizierung [2]:

- *I* Teilwandläsion (Mukosa/Submukosa) ohne Mediastinal- oder subkutanes Emphysem,
- *II* Vollwandläsion mit Mediastinal- oder subkutanem Emphysem, aber ohne Beteiligung von Ösophagus oder Mediastinalgewebe,
- *III A* Vollwandläsion mit Herniation von Ösophagus oder Mediastinalgewebe, jedoch ohne Ösophagusverletzung oder Mediastinitis,
- *III B* Vollwandläsion mit Ösophagusverletzung oder Mediastinitis,
- *IV* ausgedehntes Weichteiltrauma oder Frakturen der trachealen Knorpelringe.

Die klinische Präsentation zeigt sich meistens innerhalb von drei Tagen nach der endotrachealen Intubation und umfasst häufig ein subkutanes Emphysem im Gesicht und im oberem Thorax sowie Husten. Symptome können von leichten Atembeschwerden bis hin zum akuten respiratorischen Versagen reichen, abhängig von der Schwere der Läsion und einem möglichen ein- oder beidseitigen Pneumothorax. Teilwandläsionen hingegen verlaufen oft asymptomatisch. Bei beatmeten Patienten kann sich eine ITI schleichend entwickeln, insbesondere wenn die Cuff-Manschette die Läsion überdeckt [4]. Alternativ kann eine plötzliche, schwere Form auftreten, mit massivem Pneumomediastinum, Spannungspneumothorax und erschwerter Beatmung. Je nach Mediastinalbeteiligung können ein Pneumoperikard, eine Angina oder ein kardiogener/hypovolämischer Schock auftreten. Hämoptysen oder Pneumoperitoneum sind selten beschrieben [5].

Ursachen trachealer Läsionen sind meist multifaktoriell. In einem Fall kam es nach einer intratrachealen Laserung bei liegendem Tracheal-Stent etwa 1 h nach unauffälliger Extubation zu einer plötzlichen Tracheaverlegung mit letaler Hypoxie (nichtpublizierte persönliche Beobachtung).

Ähnlich zu unserem Fall beschrieben Vetrugno et al. eine postoperative Trachealläsion nach Schulteroperation unter Allgemeinanästhesie mit verzögertem subkutanem und mediastinalem Emphysem, vermutlich infolge von Husten und Kopfbewegungen bei liegendem Tubus [6].

Mechanische Schwierigkeiten bei der Platzierung eines Doppellumentubus oder der Einsatz eines Führungsdrahts gelten als weitere Risikofaktoren [7]. Kübler et al. zeigten in einem Fallbericht, dass Verletzungen durch die beim Entfernen eines stark gekrümmten („Hockeystick“-förmigen) Führungsstabs auf die Tubusspitze übertragene Kraft entstehen können [13].

Kotoda et al. bestätigten diesen Mechanismus in einer In-vitro-Studie: Mit zunehmender Angulation steigen die auf Trachea und Larynx übertragene Kraft und damit das Verletzungsrisiko, während ein bogenförmig modellierter Führungsstab dieses Risiko deutlich reduziert [14].

Viele ITI bleiben unerkannt, was die Diagnostik und Behandlung verzögert und die Prognose verschlechtert. Bei Verdacht, insbesondere nach einer Intubation, sind CT und Bronchoskopie essenziell [1, 2, 7]. Während das CT Trachealverletzungen und Begleitschäden detailliert darstellt, bleibt die flexible Bronchoskopie der Goldstandard zu genauer Lokalisation und Charakterisierung der Verletzung. Eine vollständige Diagnostik erfordert beide Verfahren [1, 2, 7]. Die möglichen Therapieoptionen in Abhängigkeit vom Schweregrad der Verletzung und der führenden Klinik sind in Tab. 1 zusammengefasst [7, 9–11]. In einer Berliner Fallserie konnte bei drei Fällen einer Trachealläsion, wie in unserem zweiten Fall, mit einem konservativen Vorgehen eine vollständige Erholung erreicht werden [11].

**Tab. 1:** Schwergrad der ITI und Therapieoptionen

Therapie	Schwergrad der ITI	Methoden
Konservativ	I–IIIa (bis 9 cm Riss)	*Beobachtung:* Ruhe, Antitussiva, Antibiotika, v. a. bei kleinen (< 2 cm) Stadium-I-Läsionen
		*Intubation:* Platzierung der Cuff-Manschette distal der Läsion, bei großen Defekten ggf. ECMO erforderlich
		*Tracheotomie:* Option bei langen Stadium‑I und Stadium-II-Läsionen zu Druckreduktion und Heilungsförderung
		*Fibrinklebung:* endoskopisches Verschließen mit hoher Erfolgsrate in spezialisierten Zentren
Endoskopische Therapie	IIIa und IIIb	Als Alternative zur chirurgischen Versorgung: vorübergehende Platzierung eines bedeckten Metall- oder Silikon-Stents (4 bis 8 Wochen) zu Defektüberbrückung und Spontanheilung
Chirurgische Therapie	Große IIIA-Läsionen	Frühzeitige Operation innerhalb von 48–72 h:
	IIIB-Riss mit Gefäß- oder Ösophagusbeteiligung	*Offene Verfahren:* Rechtsseitige posterolaterale Thorakotomie (für mittlere/untere Trachealverletzungen) oder Zervikotomie (für obere Drittel, ggf. mit Sternotomie)
	Stadium IV oder mit begleitender Mediastinitis	*VATS:* minimalinvasive Ansätze wie rechtsseitige Thorakoskopie oder der videoassistierte transzervikale-transtracheale Zugang
		*Endotracheale Reparatur:* intraluminale endoskopische Nahttechnik mit reduziertem Trauma und geringeren postoperativen Schmerzen

Risikofaktoren für ITI sind Notfallintubationen, Doppellumentuben, spezielle Atemwegshilfen, Unerfahrenheit des Intubierenden, weibliches Geschlecht des Patienten, höheres Alter und tracheale Anomalien [2]. Darüber hinaus haben wir weitere potenzielle Einflussfaktoren in Betracht gezogen; z. B. unterschiedliche Tuben (RAE vs. Rüsch) in unseren Fällen. Der Cuff-Druck wurde unmittelbar nach der Intubation gemessen und lag unter 30 mm Hg. Es ist bekannt, dass endotracheale Tuben mit supraglottischer Absaugung eine Hyperämie an der Cuff-Stelle und möglicherweise Trachealverletzungen verursachen können [12]. Die in unseren Fällen verwendeten Tuben sind bislang jedoch nicht mit solchen Komplikationen in Verbindung gebracht worden. Ein Hauptdiskussionsthema ist die Wahl der optimalen Tubusgröße. Bei einer In-vitro Studie an frischen Leichen konnte gezeigt werden, dass nur eine Tubusgröße größer „als normal“ die Gefahr einer Trachealruptur bei gleichem Cuff-Druck des Tubus verdreifachte [8].

In unserer Klinik gilt als Standard ein Tubus der Größe 7,5 für Frauen und 8,0 für Männer. Experimentelle Studien zeigen jedoch, dass das Geschlecht allein ein unzureichender Parameter für die Tubusgröße ist. Stattdessen sollte die Körpergröße des Patienten als entscheidender Faktor berücksichtigt werden. Laut der Studie von Sudhoff et al. sollten kleinere Patienten entsprechend kleinere Tuben erhalten, um das Risiko von Atemwegskomplikationen zu minimieren [8].

Im ersten Fall zeigte die Patientin einen starken Hustenreiz. Der dabei entstehende hohe intrathorakale Druck (bei möglicherweise temporär geschlossenem Atemwegsventil) könnte eine bestehende Läsion verschlimmert und zur ausgeprägten Symptomatik beigetragen haben. Diese Überlegungen verdeutlichen die Notwendigkeit einer detaillierten Analyse der individuellen perioperativen Faktoren, um das Risiko für ITI besser einzuschätzen und mögliche präventive Maßnahmen identifizieren zu können.

## Fazit für die Praxis

- Tracheale Läsionen können auch bei scheinbar völlig komplikationsloser Intubation und problemloser Anästhesieführung auftreten und können schwerwiegende Folgen haben.
- Im deutschsprachigen Raum sind wenige Fälle trachealer Läsionen bei einer Routine-Anästhesie publiziert. Unklar ist, ob dies ein Publikationsbias oder eine extrem niedrige Inzidenz ist. Angesichts einer Inzidenz einer trachealen Läsion bei Anästhesie von 1:20.000 bis 1:75:000 und etwa 17 Mio. jährlich durchgeführten Anästhesien in Deutschland entspräche dies eigentlich etwa 200–800 Patienten mit diesen Läsionen pro Jahr.
